# Vector-based navigation in desert ants: the significance of path-integration vectors

**DOI:** 10.1007/s00359-024-01725-2

**Published:** 2024-12-03

**Authors:** Beatrice Voegeli, Stefan Sommer, Markus Knaden, Rüdiger Wehner

**Affiliations:** 1 Canton of Zurich, Office of Landscape and Nature, Zurich, Switzerland; 2 Department of Evolutionary Biology and Environmental Studies, University of Zurich, Zurich, Switzerland; 3 Department of Evolutionary Neuroethology, Max Planck Institute for Chemical Ecology, Jena, Germany; 4 Brain Research Institute, University of Zurich, Zurich, Switzerland

**Keywords:** Insect navigation, Path integration, Shortcutting, Vector-based navigation, *Cataglyphis* ants

## Abstract

In the longstanding discussion of whether insects, especially central place foragers such as bees and ants, use metric representations of their landmark surroundings (so-called “cognitive maps”), the ability to find novel shortcuts between familiar locations has been considered one of the most decisive proofs for the use of such maps. Here we show by channel-based field experiments that desert ants *Cataglyphis* can travel such shortcuts between locations (defined by memorized goal vectors) just on the basis of path integration. When trained to visit two spatially separated feeders A and B they later travel the hitherto novel route A→B. This behavior may originate from the interaction of goal vectors retrieved from long-term memory and the current vector computed by the continuously running path integrator. Based on former experiments, we further argue that path integration is a necessary requirement also for acquiring landmark information (in form of learned goal-directed views). This emphasizes the paramount importance of path integration in these central place foragers. Finally we hypothesize that the ant’s overall system of navigation consists in the optimal combination of path-integration vectors and view-based vectors, and thus handles and uses vectorial information without the need of constructing a “vector map”, in which vectors are linked to known places in the environment others than to the origin of all journeys, the nest.

**Supplementary Information:**

The online version contains supplementary material available at 10.1007/s00359-024-01725-2.

## Introduction

Path integration — a means of navigation, by which an animal uses egomotion cues to keep a continually updated record of its direction and distance from the starting point of a journey — is a basic navigational routine widespread in the animal kingdom. Experimental studies of this routine have been performed in both the field and the laboratory especially in insects, crabs, and spiders, but also in birds and mammals including humans (for reviews, see e.g., Chap. 4 in Gallistel 1990; Chap. 3 in Papi 1992; Chap. 3 in Healy 1998; Chap. 3.8 in Golledge 1999; Chaps. 1 and 3 in Jeffery 2003; Heinze et al. 2018; Chap. 5 in Wehner 2020; and Chap. 5 in Mallot 2023).

In *Cataglyphis* desert ants path integration is of major importance. This is already borne out by the multiple compass and odometer systems, which the foraging ants employ for path integration — a navigational routine that keeps running all the time while the animals move about in their nest surroundings. In detail, *Cataglyphis* continually derives directional information from skylight cues (Wehner 1994; Wehner and Müller 2006), the geomagnetic field (Fleischmann et al. 2018a), and the direction of the wind (Wehner and Duelli 1971; Wolf and Wehner 2000; Müller and Wehner 2007), while it simultaneously gains distance information from self-induced visual flow (Ronacher and Wehner 1995; Pfeffer and Wittlinger 2016) and stride integration (“step counting”, Wittlinger et al. 2006). The data obtained these ways about the animal’s angular and linear components of movement are integrated into a continuously updated path integration vector (home vector) constantly pointing back to the start of the foraging journey, the nest entrance (home).

A striking example of how decisively *Cataglyphis* depends on navigation by path integration is provided by the salt-pan ant *Cataglyphis fortis*. This species inhabits the vast flat terrains of the North African chotts, which are almost free of any landmark cues that could be used in long-distance navigation. When the foraging ants venture out from their underground nest for distances of more than one hundred meters (Wehner 1987; Bühlmann et al. 2014; Huber and Knaden 2015), they almost exclusively depend on path integration. Their dependence on this navigational routine is so strong that their path integrator is reset to zero-state only when the animals have returned to the nest and have finally entered it. When instead they are displaced from the food site directly to the nest entrance with all nest defining cues available — such as nest odor, small nest surrounding landmarks, and nestmates rushing in and out the entrance hole — they do not enter the nest but run off their home vector by unhesitatingly hurrying away from the nest in the feeder-to-nest direction (Knaden and Wehner 2006). It is only when they are inside the nest either by having actively returned to it from their foraging journeys or by being forced by the experimenter to enter it that their path integrator is reset to zero-state.

Usually, path integration (PI) is considered a low-level navigational process confined to the animal’s working memory (for a recent reference, see Mallot 2023, p. 144). However, in *Cataglyphis* PI plays a much more important role than merely bringing the animal back to the start of its foraging journey. Even if the ant has reached home and reset its path integrator, information about the home vector of the last trip is not lost but preserved in long-term memory. When starting a new foraging journey the ant may retrieve this home vector from memory and use it, inverted in sign, as a reference vector leading the forager back to a previously visited food site. Again, this may happen in landscapes that are free of landmarks, and it may happen several days after the ant has returned from the journey to the previous site. It was already in one of the very first experiments performed in studies of *Cataglyphis* navigation that the animals when starting a foraging journey strictly followed a previously obtained PI food vector, even if sets of familiar landmarks pointed in a different direction (Wehner 1970; see also Wehner et al. 2002; Bolek and Wolf 2015).

During its lifetime a *Cataglyphis* ant may visit and repeatedly return to more than one feeding site, and hence may acquire more than one food vector (e.g., Wehner et al. 1983). In other words, within an origin (nest) centered frame of reference an ant may acquire PI coordinates of several places within its nest environs. What use could the animal make of this vector information? Here we address this question by designing a particular channel setup to train *Cataglyphis* ants to visit two feeding sites and thus to simultaneously acquire food vectors of two places (PI coordinates x_1_/y_1_ and x_2_/y_2_; nest coordinates 0/0), and subsequently test whether the ants are able to travel novel shortcuts between the two places. We show that the ants can accomplish this task by exclusively employing PI vector information. We further argue that even the additional acquisition of landmark information requires the aid of a continuously working path integrator, and thus emphasize the basic and paramount importance of path integration in the ants’ overall system of navigation.

## Materials and methods

The experiments were done between late June and early September 2005 in a vegetation-free salt-pan area near the settlement of Mahrès, Tunisia (34° 32’ N, 10° 32’ E). They were usually carried out daily, from 8:30 to 18:00 (local standard time), except for overcast or windy days.

### Experimental setup

**Fig. 1:**
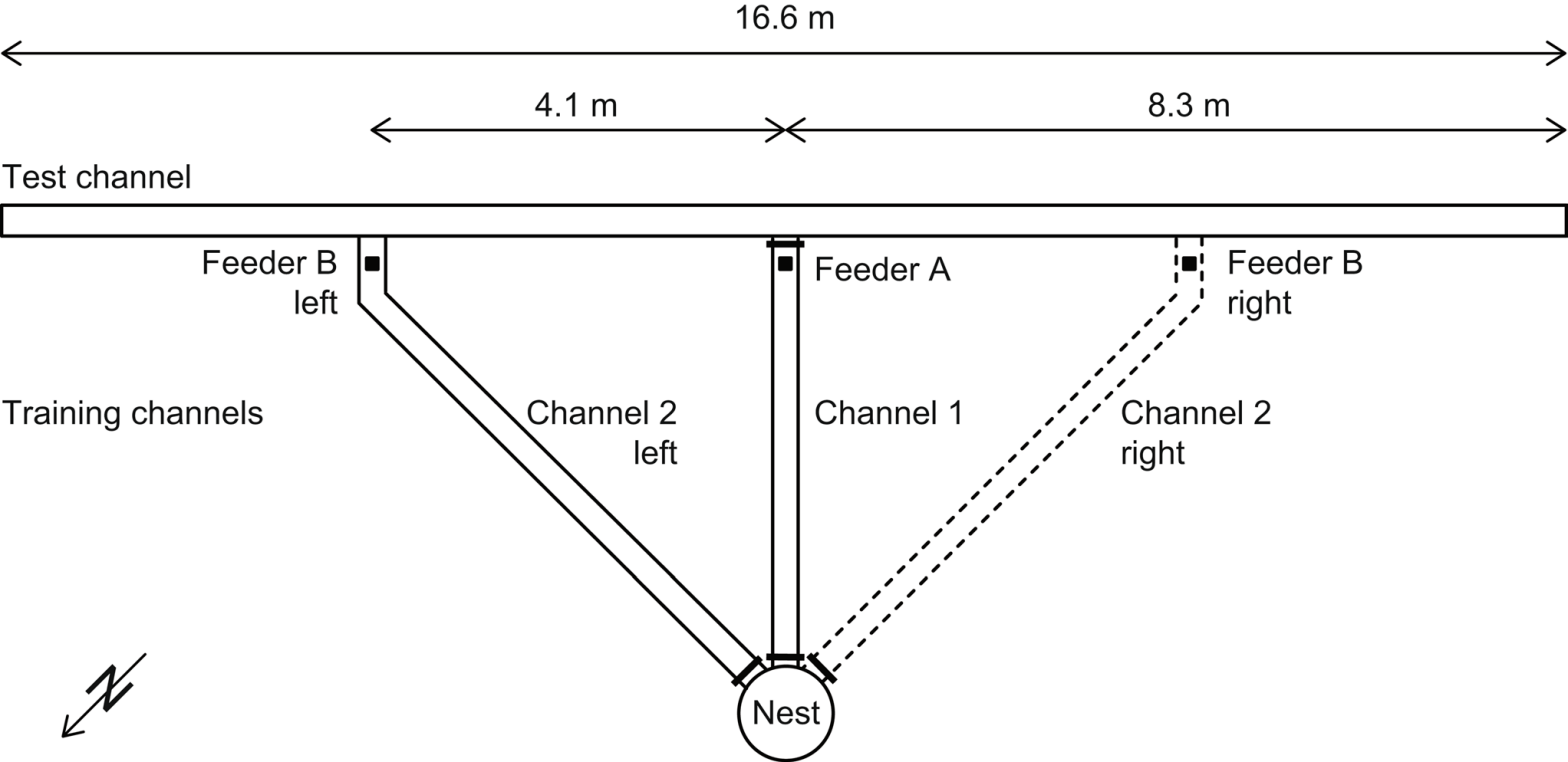
Schematic drawing of channel setup (not to scale). Ants were trained to forage at feeders A and B (black squares) within channels 1 and 2, respectively. During training of a given ant, feeder B was to either the left or the right of feeder A. The distances from the nest to feeders A and B were 4.5 m and 5.7 m, respectively. Small thick black bars at the nest, and between feeder A and the test channel, represent metal plates (shutters) that could be removed to open the channel entrances. At any one time, only one channel entrance was open. The circle around the nest represents a barrier that ensured that the ants would enter the training channels. Details on the training and testing procedures are given in the text

Foraging ants were trained and tested in an array of linear aluminum channels connected to a subterranean nest (Fig. 1). To guide the foragers into the channels, the nest entrance was enclosed by an inverted, bottomless plastic bucket with holes leading into the training channels. These holes could be opened and closed. All channels had a cross section of 7 × 7 cm^2^. Thus, ants running in the middle of a channel could see the sky under a strip-shaped window with a width of about 53° and thereby access compass information necessary for path integration; yet the view to the surrounding landscape was blocked. To mimic the ants’ natural running surface, the channel floors were covered with sand. Moreover, to hinder the ants from escaping, the interior walls were covered with clear adhesive foil. Because the channel walls were uniformly gray, they lacked any cues that could have been used by the ants for navigation. To prevent ants from exploring the channels outside the experimental sessions, the channels were set up every morning and taken down every evening.

### Experimental procedures

Ants were trained to forage at two feeders that were placed in different channels (differing by an angular amount of 45°) at either 4.5 m (feeder A) or 5.7 m (feeder B) from the respective channel entrance (Fig. 1). For selective training, only one channel entrance was open at a time. The feeders consisted of plastic cups lowered to ground level through holes that were cut into the channel floors, so that the feeders were hidden from the view of the approaching ants. The feeders usually contained biscuit crumbs (occasionally soaked in melon juice) and sometimes small pieces of dried tuna. To prevent ants from foraging unnoticed, the feeders were constructed as traps that could be left only via a twig that was added (and removed) by the experimenter. Therefore, the feeders were checked for trapped ants approximately every 10 min. Ants that were trapped for the first time received an individual color code (one dot of acrylic paint on the alitrunk and two dots on the gaster). Individually marked ants then had to complete ten training runs. The first four runs were to feeder A in channel 1, the next four runs to feeder B in channel 2, and the last two runs again to feeder A. As viewed from the nest, feeder B was located at an angle of 45° to feeder A either to the left or the right (hereafter referred to as L-training or R-training; see solid and dashed channel signatures in Fig. 1). Only ants that had completed their ten training runs were used in the critical tests.

Ants were tested one by one. When a trained ant re-entered channel 1, the channel entrance near the nest was closed (to prevent other ants from entering), the feeders were removed, and the entrance to the test channel was opened. In search of the now missing feeder A, the ant soon entered the test channel. The entrance to this channel was immediately closed. Subsequently, the ant continued to search for food by running back and forth in the test channel. To characterize this search behavior, we recorded the first ten U-turns of each ant (to the nearest 5 cm) using a tape measure that was laid out along the test channel, and noted the time of day of each test run (the data are provided in supplementary Table S1).

In control experiments individually marked ants were trained to only one feeder, feeder A, four times before they were tested. The testing was done as for ants that had been trained to two feeders.

Within the different experiments, data were collected with ants taken from up to four nests. Because the search behavior of ants from different nests was similar in a given experiment, the data were pooled across nests. At each nest, individual ants were tested only once. Occasionally, ants aborted the test run before they had completed ten U-turns (some ants escaped or found food occasionally drifted there by the wind, others were attacked by robber flies or distracted by other ants that had found their way into the test channel). Aborted runs were excluded from the analyses.

### Data analysis

First, we converted the distance values of all U-turns into distances from the starting point. For each test run, we then analyzed two criteria: (i) the direction of the ant’s first U-turn relative to the starting point and (ii) the distribution of the ants’ search densities. Criterion (i) indicated whether ants that had been trained to two feeders but had failed to find feeder A (because it had been removed) continued to search for food in the direction of feeder B. Ants that in the control experiment had been trained only to feeder A were expected to continue their searches with equal probability to the left and right of the starting point. Criterion (ii) would show whether ants trained to the two feeders but having failed to find feeder A biased their searches toward the location of the (removed) feeder B. Ants that had been trained only to feeder A were expected to search symmetrically around the starting point, that is, around the removed feeder A.

The search density distributions (criterion ii) were computed as follows: First, we divided the length of the test channel into 10-cm bins. The central bin, the one around the starting point, extended from − 5 cm to + 5 cm. Using a MATLAB routine, we then counted how often a given ant entered each bin. Finally, we transformed the counts so that they summed up to a search density of 1.0 (100%) per ant. Normalizing the counts ensured that each ant was represented equally in aggregated graphs comprising the search density of multiple ants.

Whenever ants and bees have a limited view of the pattern of polarized light in the sky, as is the case in the channel experiments, the accuracy of their compass readings is impaired. These channel-induced inaccuracies have been studied in detail and have finally led to an understanding of how bees and ants use polarized skylight as a compass cue (Rossel and Wehner 1984; Fent 1985; Wehner and Müller 2006). In the present case, these “channel errors” (the channel-induced compass errors) depend on the width of the skylight window seen by the ants in the channel and on the running direction of the ants relative to the solar meridian, that is, on the geographical orientation of the channel and the time of day. As a consequence, the ants’ perception of spatial relations within an array of channels, as in our setup, might be distorted. Specifically, ants might misjudge the distance between feeders A and B.

To account for the navigational error introduced that way by our channel setup, we used data about channel-induced compass errors as reported by Müller (1989 and Fig. 19 therein). First, we estimated the ant-subjective channel orientations for 30-min intervals (Table 1). For each interval, we then drew the corresponding (i.e., distorted) channel setup and estimated the ant-subjective position of feeder B. When this position lay outside the test channel, we projected it vertically onto the test channel. Next, we compared the search density distribution of two groups of ants: (i) ants whose predicted perception of the distance between feeder A (the starting point of the ants’ search) and feeder B was more than 10 cm shorter than the true distance (underestimation group), and (ii) ants whose predicted perception of the distance between feeders A and B was more than 10 cm longer than the true distance (overestimation group). We excluded those few ants, whose predicted perception of the distance between A and B lay within the range of +/- 10 cm of the true distance. Finally, because we were interested in the ants’ search effort regarding feeder B (rather than feeder A), we subtracted from the acquired search density distributions of ants that had been trained to both feeders the search density distribution of ants that had been trained to only feeder A.

**Table 1:** Estimated ant-subjective channel orientations (°) and distances (m) between two feeders for different times of day. Channel orientations are relative to North (0°). For training channels, they are given in the nest-to-feeder direction; for the test channel, they are given in the direction of the channel’s right-hand segment. Training channel 2 with feeder B was either to the left or to the right of training channel 1 with feeder A. Estimates are based on data reported in Müller (1989); true values (the orientation of the channels in cardinal points and the distance between feeders A and B) are given in the bottom row

Time	Test channel	Training channel 1	Training channel 2, left	Training channel 2, right	Distance from A to B, left	Distance from A to B, right
10:45	232	128	90	180	3.5	4.5
11:15	227	133	105.5	164.5	2.7	2.9
11:45	230	130	102	168	2.7	3.4
12:15	227	133	108.5	161.5	2.3	2.8
12:45	212	148	98	172	4.0	2.6
13:15	218	142	94	176	4.1	3.3
13:45	217	143	80.5	189.5	5.3	3.9
14:15	221	139	69.5	200.5	5.6	4.8
14:45	232	128	74	196	4.3	5.7
15:15	232	128	75	195	4.3	5.5
15:45	233	127	80	190	4.0	5.2
16:15	230	130	85	185	3.9	4.9
16:45	229.5	130.5	88	182	3.9	4.4
True value	225	135	90	180	4.1	4.1

## Results

### First U-turn

Of 60 test ants that had been trained to the two feeders, two thirds performed their first U-turns on the side of feeder B after having failed to find feeder A (L-training: 21 out of 31 ants, R-training: 19 out of 29 ants). By contrast, test ants that had been trained to only one feeder began the search in similar proportions to either side of the starting point (left: 8 ants, right: 9 ants).

### Search density

**Fig. 2:**
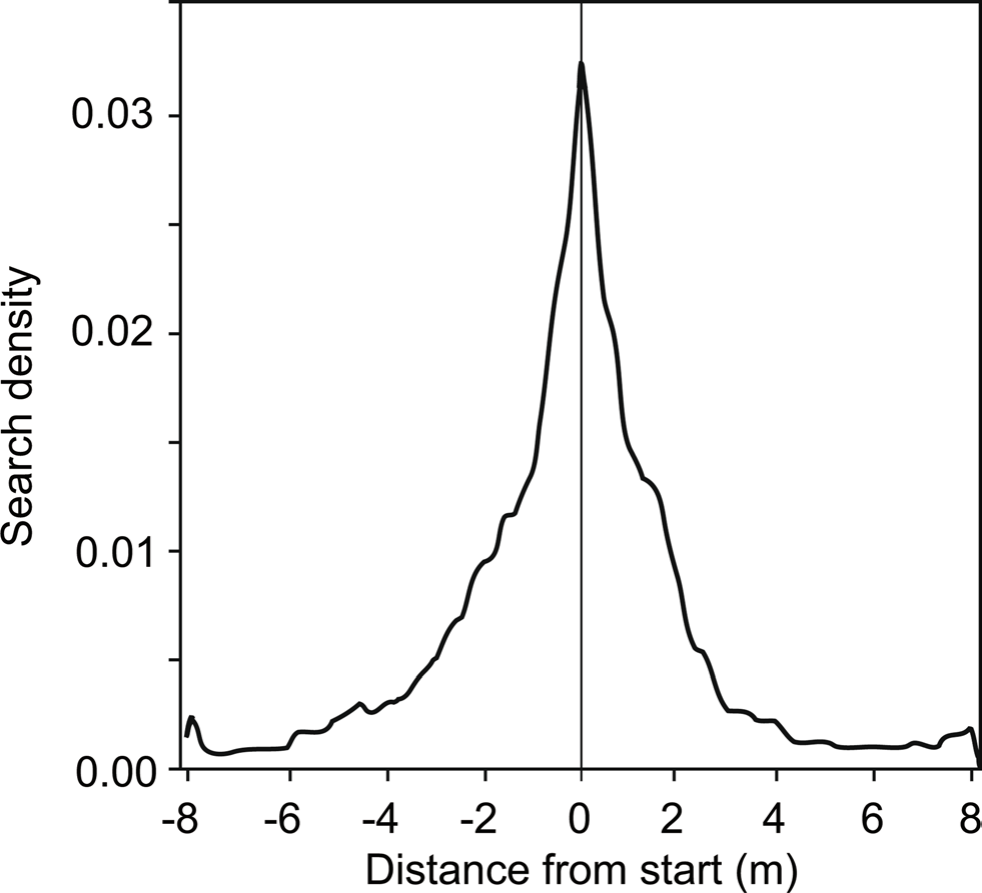
Search density distribution of 17 ants that were trained to one feeder. The feeder in the training channel was next to the starting point (vertical line) in the test channel. For tests, the feeder was removed

**Fig. 3:**
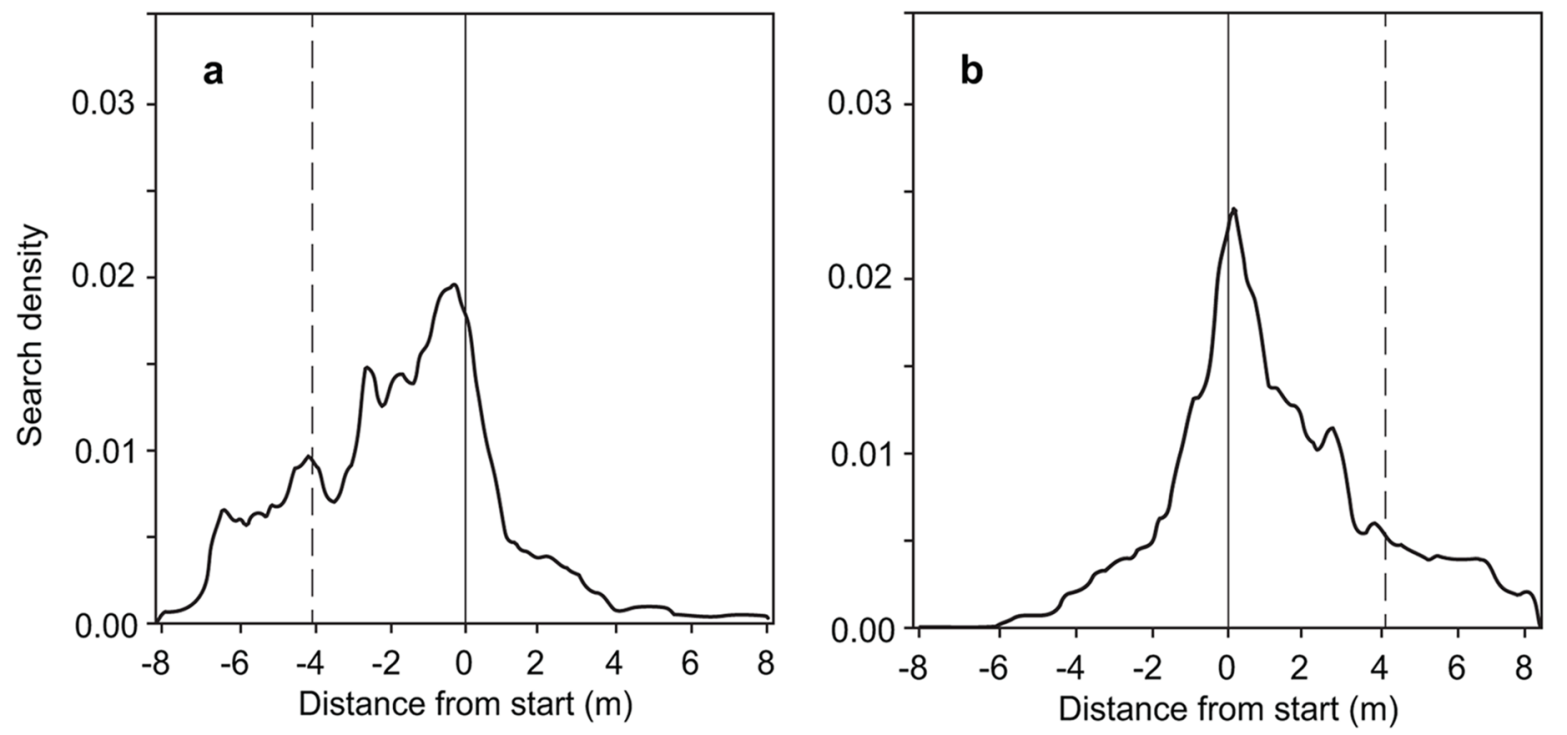
Search density distributions of ants that were trained to two feeders, A and B. During training, feeder A was at the position of the starting point (solid vertical line), and feeder B was 4.1 m away from that point (dashed vertical line). Feeder B was either to the **a** left, 31 ants, or **b** right, 29 ants, of feeder A

Ants that had been trained to one feeder searched symmetrically around the starting point (Fig. 2). By contrast, ants that had been trained to two feeders searched asymmetrically around the starting point, with a bias towards feeder B (Fig. 3). This general search pattern was unaffected by the position of feeder B (to the left or the right of feeder A). Having entered the test channel, the ants initially searched around the starting point (the location of feeder A) but then increasingly widened their searches toward the location of feeder B.

To study how the navigational error caused by the channel setup affected the ants’ search for feeder B (due to the channel alignment relative to the pattern of polarized skylight, see Table 1), we first split the ants into two groups (those who were predicted to underestimate the true distance between feeders A and B, and those who were predicted to overestimate it) and then subtracted from the resulting distributions the search density distribution of ants that had been trained only to feeder A. The search peaks were closer to the ant-subjective positions of feeder B than to the true position of that feeder. This was the case independently of whether feeder B was to the left or to the right of feeder A, and whether the ant-subjective position of feeder B was on the near side of the true position (relative to the starting point of the searches; underestimation group) or beyond (overestimation group).

As these systematic deviations of the ants’ search peaks from the true position of feeder B correspond to the expected deviations caused by the limited view the channel-bound ants have of the sky, these systematic “compass errors” can be considered the only source of the deviations from the 4.1-m mark. More importantly, however, the fact that at all the ants shift their searches significantly from the position of feeder A to that of feeder B is clear evidence that they have memorized the locations of both feeders, and that in particular situations they can flexibly head for either location.

## Discussion

The longstanding discussions of how insects, especially central place foragers such as bees, ants and wasps, find their way when moving about in their foraging terrain, finally revolve around two major hypotheses. According to one hypothesis (map-based navigation) insects use unified metric representations of their nest surroundings, so-called “cognitive maps” (Menzel et al. 2005; Cheeseman et al. 2014a with commentaries by Cheung et al. 2014; Cheeseman et al. 2014b; for historical reflections on the “map debate” see Dhein 2023 and commentary by Wehner et al. 2023). According to the other hypothesis (vector-based navigation) the insect’s navigational system is more decentralized and operates with vector-based information (Wehner et al. 1990; Dyer et al. 2002; Cruse and Wehner 2011; Hoinville and Wehner 2018; Patel et al. 2024; Wehner 2024). It has usually been argued that novel shortcutting, that is, the ability to travel a new direct route between two familiar places, is the definitive marker of a cognitive map (e.g., Tolman 1948; Bennett 1996). Here we show by channel-based field experiments that desert ants can construct novel shortcuts between known feeding places just on the basis of path integration (PI), as the channel device prevents the animals from viewing any potentially known landmark within their foraging terrain. The present study is actually the first demonstration of this ability in any insect. The main result and its interpretation has preliminarily been published in Wehner (2020, p. 182 and Fig. 5.8 therein).

**Fig. 4:**
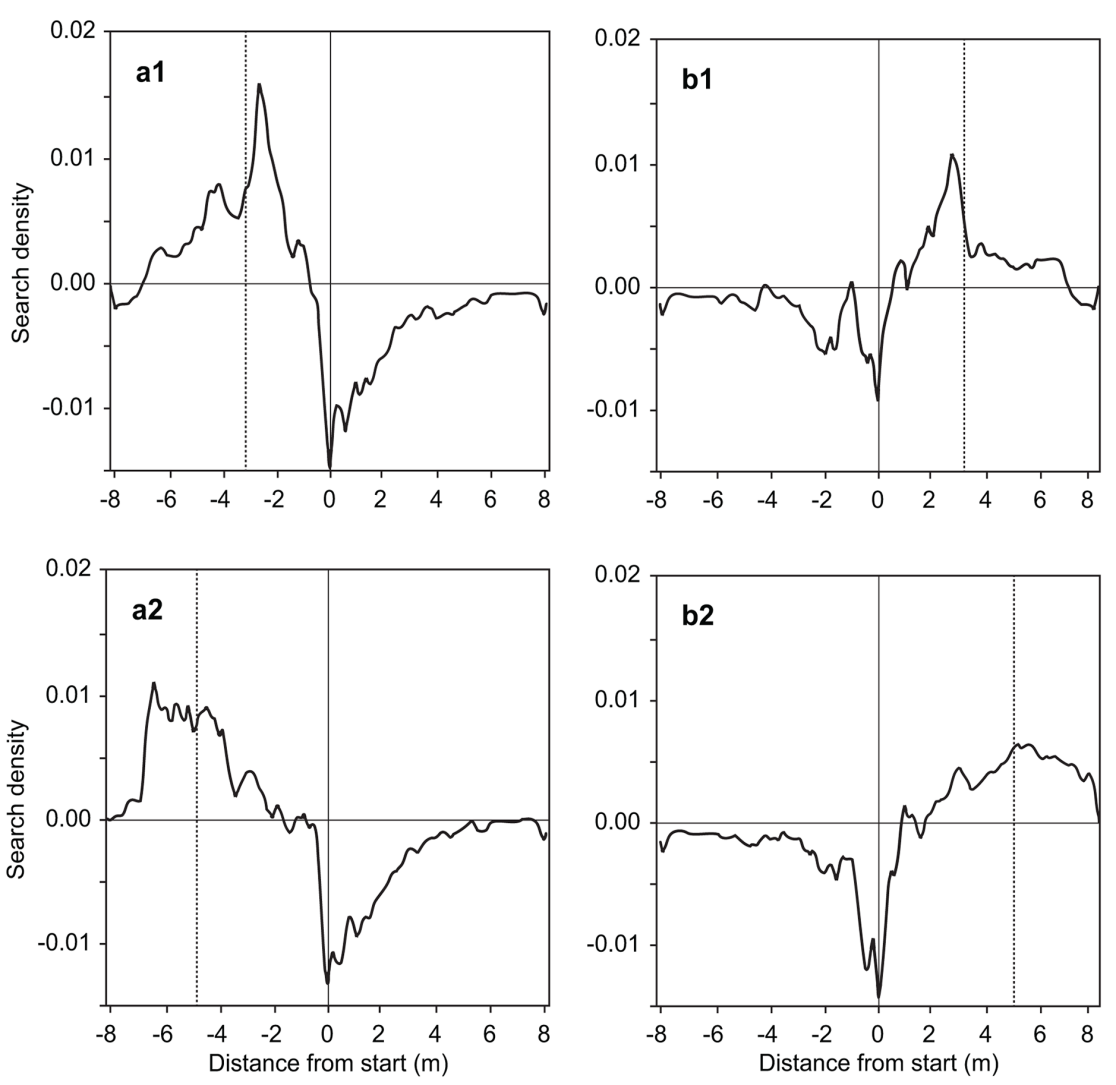
Relative search density distributions of ants that were trained to two feeders, A and B, at different times of day. During training, feeder B was either to the left (**a1** and **a2**) or to the right (**b1** and **b2**) of feeder A. During tests, both feeders were removed. Due to the channel-induced “compass errors” (see Material and Methods) the ants were expected to either underestimate (**a1** and **b1**) the true distance between the starting point (solid vertical line) and the position of feeder B (dotted vertical line) or overestimate it (**a2** and **b2**). The relative distributions were computed by subtracting from the original distributions the distribution of ants that were trained to only feeder A (cf. Figure 2). That is, positive (negative) search densities mean that the ants searched a given channel segment more frequently (less frequently) than ants trained to only one feeder. **a1** L-training, 16 ants; **a2** L-training, 11 ants; **b1** R-training, 15 ants; and **b2** R-training, 11 ants

**Fig. 5:**
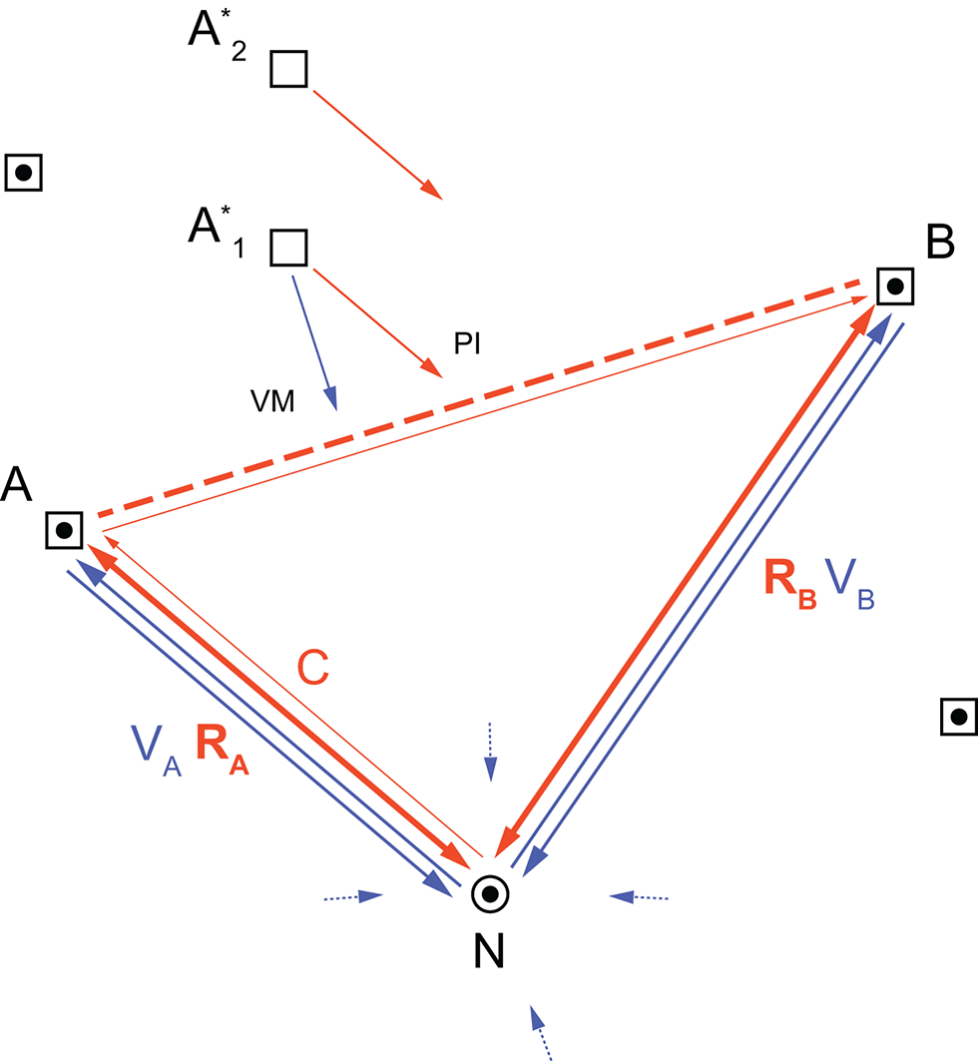
The flexible use of path-integration vectors in desert ant navigation. Ants departing from the nest, N, visit two habitual feeding sites, A and B. As a result, they have acquired (i) the path integration vectors *R*_*A↕*_ and *R*_*B↕*_ (depicted by the double-headed bold red arrows) stored in long-term memory and used as home vectors and food vectors, and (ii) view-defined routes *V*_*A↑↓*_ and *V*_*B↑↓*_ (depicted by the blue arrows). The small dotted blue arrows indicate the nest-centered views acquired by means of path integration during learning walks. The thin red arrow, *C*, marks the current path integration vector continuously running in working memory while the ant is on its journey N→A→B. The novel shortcut taken from A to B, which is exclusively determined by the ant’s path integrator (by matching *C* to *R*_*B*_) is indicated by the dashed red line. The same rationale would apply if the ant in addition frequented other places (see further square symbols with dot). Ants displaced from A to A* may return from these novel places by using their path-integration (PI) routine and — at places where view matching is still effective (at A*_1_ but not at A*_2_, see text) — view-matching (VM) routine. At A*_1_ they then may steer various kinds of intermediate courses, depending on the particular reliabilities of the PI (red) and VM (blue) vectors. The core of the figure is taken from Fig. 7.14 in Wehner (2020)

In the present experimental setup *Cataglyphis* desert ants are trained in channels to frequently visit two feeders and later tested for their ability to travel a novel shortcut from one feeder station to the other. Based on the ants’ behavior in the channel array (Fig. 1) we now discuss what the results obtained that way (Figs. 2, 3 and 4) would mean for freely walking ants trained and tested in their open nest environment (Fig. 5).

Before we start the discussion, let us make two remarks. First, during training to a food site *Cataglyphis* acquires a PI “food vector” leading it to that site. This food vector is stored in long-term memory, from where it can be recalled hours or even days later. It is always the 180° reversal of the previously acquired “home vector” (see double-headed bold red arrows in Fig. 5). Evidence for the latter statement comes from open-jaw experiments, in which the ant’s point of arrival (at the food site) does not coincide with the ant’s point of departure (from a release site), because before departure the ant is displaced from the food site to a near-by location, from which it can return to the nest only by a curved rather than the direct route (Wehner et al. 2002). Even if this open-jaw procedure is repeated dozens of times, the ant never learns to return from the release site directly to the nest. It recalibrates its home vector by a certain angular amount, but coincidentally the same recalibration occurs with the food vector paid out during subsequent foraging runs (Wehner et al. 2002), so that the relation food vector = home vector +/- 180° always holds.

Second, how does the PI system operate while the ant moves toward a familiar food site? We hypothesize that when setting out for a foraging run to that site, the ant activates the respective food vector as a reference vector *R* (Fig. 5: bold red arrow) and loads it down from long-term memory into its working memory. Subsequently, while on the move it continually compares this reference vector with the continuously updated current PI vector *C* (Fig. 5: thin red arrow) and walks until both vectors match (Collett et al. 1999; Collett and Collett 2000; Wehner 2020, p. 181f).

With these remarks we return to the results of the present study and discuss how the ants could have used their path integration system to perform the novel shortcut. During training to food sites A and B the ants have acquired two food vectors stored in long-term memory. When they start a new foraging journey these food vectors are activated so as to potentially getting employed during the journey. In working memory, they may then serve as reference vectors, *R*_*A*_ and *R*_*B*_, respectively. Now assume that the animal has arrived at site A, which had provided food most recently, but is presently devoid of food. It then starts to search, though unsuccessfully, in ever widening loops (back-and-forth movements in the channel) around that place. As the search proceeds, the probability of finding food at A and thus the weight of vector *R*_*A*_ decreases. Finally, when the weight of *R*_*B*_ leading to the less recently visited food site B outweighs that of *R*_*A*_, food vector *R*_*B*_ becomes dominant. The ant then moves until the current vector *C* matches *R*_*B*_ (Cruse and Wehner 2011; Wehner et al. 2014).

One could also hypothesize that when being unsuccessful at place A the ants would compute the shortcut A→B by performing vector subtraction of the stored vectors *R*_*A*_ and *R*_*B*_ (Collett and Graham 2004) without any reference to the current state of the path integrator. This could include resetting the path integrator. However, according to our present knowledge resetting of the path integrator to zero-state occurs only when the ants have re-entered their colony (Knaden and Wehner 2006). Hence, we favor the hypothesis mentioned above that when the ants are inclined to move from A to B, they just load down *R*_*B*_ into working memory. With their continuously running path integrator they would then directly be led to the desired destination. When foraging in their natural environment the ants when starting for a new journey may activate all stored food vectors and thus keep them ready for being loaded down into working memory according to the weights of the respective feeding sites at any particular time. The weight attributed to a given feeding site may depend on the distance of the food source from the nest, the quality of the food, the time passed since the last visit, etc.

Patel et al. (2024) in studying path integration in bumblebees (*Bombus terrestris*), which walked over short distances in a laboratory arena, have addressed questions of how PI vectors stored in long-term memory may be used in navigation. Bees with their wings clipped could move freely in the arena and finally return from a feeder placed in the center of the arena to the hive at the periphery (Patel et al. 2022). Compass information was provided by an overhead linear polarizer, that is, a zenith-centered e-vector. In particular experimental paradigms rotating the e-vector by 90° caused the position of the feeder to be shifted by 90° from its habitual position. Based on such and further experiments, the authors conclude that the bees can store home vectors in long-term memory, and then perform vector computations with these food vectors in the way described in the previous paragraph as an alternative to the hypothesis proposed in the present account.

Let us now address the question of how the PI-based shortcutting could be embedded in a larger navigational context in which the animals had access also to external cues such as landmarks. Could they then associate landmark views perceived and learned around locations A and B with the nest-centered PI coordinates of these locations? One way for tackling this question would be to train the animals in an experimental setup similar to the one applied here but in addition providing the animals with conspicuous landmark panoramas defining A and B. Now assume that the animals, after they had learned these panoramas, were just going to set out for a new foraging run, but were displaced from the nest to either A or B (“zero-vector animals”). If they then traveled directly to B or A, respectively, they would have used the learned spatial relation of A and B. Such experiments have not yet been done under well controlled conditions in either ants or bees. In *Cataglyphis fortis*, preliminary experiments (S. Schwarz and R. Wehner, unpubl.) yielded negative results (for open-field experiments with honey bees, see Dyer 1991). Moreover, when at a landmark-defined place a stored PI vector and the still running current PI vector point in different directions, the ants always follow the dictates of the latter (Sassi and Wehner 1997; Collett et al. 2003). Difficulties in associating landmark information with PI coordinates might arise from the fact that the ants use landmark information in form of goal-directed views rather than place-defining views (Müller and Wehner 2010; Graham et al. 2010; Fleischmann et al. 2017).

Panoramic goal-directed views can be acquired and stored in long-term memory not only from different locations around a goal but finally also along entire routes leading to a goal (Kohler and Wehner 2005; Sommer et al. 2008; Mangan and Webb 2012; Schwarz et al. 2017). Hence, in navigation ants use view-based vectors (Fig. 5: blue arrows) in addition to PI vectors (Fig. 5: bold red arrows). While the home PI vector is the 180° state of the food PI vector (see above), the ants acquire different view vectors for the food run and the home run (views perceived along a route depend on the direction of travel), so that view-based vectors point in only one direction (Wehner et al. 2006; Mangan and Webb 2012). In operational terms, it is a likely hypothesis that the ant while on its way continuously compares the current views with the stored ones (Lent et al. 2010; Baddeley et al. 2012) and moves in the direction in which both views match best, that is, in which the rotational image-difference function exhibits its minimum (Zeil et al. 2003; Wystrach et al. 2011; Zeil 2023). As in general the landmark panorama will not change dramatically over relatively short distances, the difference function may exhibit a minimum, though a shallower one, even at places where the ant has not been before (Fig. 5: displacement of the ant from A to A_1_*; for experimental evidence, see Narendra 2007; Narendra et al. 2013; Zeil et al. 2014; Fleischmann et al. 2018b). This ability to return home even from unfamiliar places renders the view-matching strategy a rather powerful means of navigation.

In conclusion, shortcutting and returning from novel places — two behaviors always considered major proofs of an animal’s use of a cognitive map — can both be understood on the basis of the vector-based navigational strategies described here. We even hesitate to speak of a “vector map”, as we have no indication yet that stored PI vectors are anchored at familiar places other than the origin, the nest. All navigational performances observed until now in desert ants can be interpreted and simulated by the optimal combination of path-integration (PI) and view-matching (VM) routines with “optimal” meaning that the path-integration vectors and view-based vectors are combined according to how certain, that is, reliable they are at any one time and at any one place (Hoinville and Wehner 2018; Wehner 2020, p. 280f).

In evolutionary perspectives, (i) path integration — widespread in the animal kingdom — provides an animal with a continuously homeward pointing PI vector (the “current vector” in short-term memory) and thus enables the animal to directly return to the start of a foraging or any other journey. In addition, (ii) storing PI goal vectors in long-term memory allows for frequent returns to desirable destinations and, as shown here, for PI-based shortcutting. When landmarks are around, as they are in most cases even in desert ants, the PI system is used to (iii) acquire goal directed views from places to the goal and along routes leading to the goal. That way, path integration is a necessary requirement even for providing the navigational system with landmark information. A further level of complexity would be reached if (iv) PI coordinates of goals could be associated with external cues perceived at the goals and rendering the “vector map” a likely hypothesis. Note that in all these cases vectors combine known places rather than landmarks around these places. Determining vectors combining the latter, as sketched out in Wolf (2024), would certainly require some advanced methods of surveying technology and lead to the construction of a metric map.

Based on our present knowledge, and as proposed above in (iii), we hypothesize that the ants’ navigational system grounded in path integration handles and uses vector information by combining path-integration vectors and view-based vectors according to their actual reliabilities. In this view there is no need yet to incorporate these vectors into a map-like representation, a “vector map”.

As to computational models of path integration, the hypothesis of a ring attractor network has been proposed independently in the same year for laboratory rats and desert ants by Skaggs et al. (1995) and Hartmann and Wehner (1995), respectively. In the insect brain such ring-like topologies with the proper neuronal networks are represented in the central complex, where they have been studied first and most extensively in fruit flies *Drosophila* (Seelig and Jayaraman 2015; Kim et al. 2017; Hulse et al. 2021), but also in halictid bees *Megalopta* (Stone et al. 2017) and bumble bees *Bombus* (Sayre et al. 2021). Neural correlates especially for steps (i) and (ii) mentioned above are described and discussed in the central complex of *Megalopta* by Stone et al. (2017) and Le Moël et al. (2019), respectively. Moreover, based on the intensive research presently focusing on the fan-shaped body in the central complex of *Drosophila* (Lyu et al. 2022; Lu et al. 2022) hypotheses of more advanced ways from egocentered (animal centered) to geocentered (world centered) representations have been developed. What is now needed in ants, bees, and other insect navigators are carefully controlled behavioral studies scrutinizing what use the animals may actually make of the computational potentialities that are increasingly uncovered in their central complex and other forebrain neuropils.

## Electronic supplementary material

Below is the link to the electronic supplementary material.

Supplementary Material 1

## Data Availability

All data on the ants’ first ten U-turns that support the findings of this study are included within this paper and its Supplementary Information file.
