# Supplementary material for: Vector-based navigation in desert ants: the significance of path-integration vectors
Source: J Comp Physiol A Neuroethol Sens Neural Behav Physiol. 2024 Dec 3;211(2):209–20. doi: 10.1007/s00359-024-01725-2 (PMC12003618; doi:10.1007/s00359-024-01725-2)
Supplement: Supplementary file 1 — Supplementary Material 1 [file 359_2024_1725_MOESM1_ESM.pdf]

Supplementary Information

Journal of Comparative Physiology A

Vector-based navigation in desert ants: the significance of path-integration vectors

Beatrice Voegeli[1], Stefan Sommer[2], Markus Knaden[3], Rüdiger Wehner[4]

1 Canton of Zurich, Office of Landscape and Nature, Zurich, Switzerland

2 University of Zurich, Department of Evolutionary Biology and Environmental Studies, Zurich, Switzerland

3 Max Planck Institute for Chemical Ecology, Department of Evolutionary Neuroethology, Jena, Germany

4 University of Zurich, Brain Research Institute, Zurich, Switzerland

Correspondence: ruediger.wehner@uzh.ch

Table S1 Original data of the two-feeder experiment and the one-feeder control experiment. In the two-feeder experiment, feeder B during training was to either the left (L-training) or the right (R-training) of feeder A. Between the two training conditions, the tape measure was flipped so that feeder B always was at the 12.4-m mark. In the control experiment, the tape measure always started at the right-hand side of the test channel. In all experiments, the starting point of the ants' search was at the 8.3-m mark. (Note that for the analyses the data were transformed, so that in figures the starting point would be at the 0-m mark, and the right and left test-channel segment would align with positive and negative distance values, respectively.) Three-letter acronyms (first column) refer to the color code of individual ants. Time is local standard time of the ants' test run; numbers 1 to 10 are the positions (m) of the ants' first ten U-turns within the 16.6-m long test channel.

Two-feeder experiment, L-training (right channel end at 0 m; left channel end at 16.6 m)

Ant	Time	1	2	3	4	5	6	7	8	9	10
ywl	11:21	10.60	10.10	11.30	9.10	9.60	8.50	11.10	8.60	11.30	8.80
ylr	10:53	13.50	13.20	13.60	12.50	13.00	10.70	13.10	9.80	10.10	8.70
rry	11:22	11.00	9.40	12.60	8.40	12.95	7.80	11.60	10.20	13.35	8.50
rbr	11:12	15.40	10.90	11.60	10.80	15.45	8.40	9.60	8.70	11.00	10.70
rbl	11:42	8.05	11.10	10.20	11.60	10.70	11.10	10.00	11.10	8.20	8.90
wrw	11:52	8.60	7.80	11.85	8.40	11.20	9.70	15.10	13.10	15.20	13.70
wwb	11:12	8.15	9.50	7.20	9.60	7.70	8.10	5.10	13.00	12.25	15.20
yyr	12:05	10.40	5.40	8.50	8.10	11.00	7.90	8.90	4.50	10.70	8.30
wlw	10:45	6.90	11.70	11.00	11.30	10.70	11.10	10.00	10.20	8.50	11.20
yrr	11:23	8.55	7.30	10.20	5.70	9.80	5.10	9.30	8.40	9.70	8.35
ryy	16:35	9.10	7.30	13.85	7.60	11.15	10.75	13.20	2.60	3.80	1.00
wbb	16:15	9.10	8.10	8.50	8.00	8.40	7.95	13.10	9.20	13.95	11.70
wlb	16:30	8.60	8.35	9.10	7.75	8.20	7.25	11.10	7.80	8.35	8.00
wry	16:05	7.80	10.60	4.30	8.60	8.20	9.10	7.60	14.90	11.90	12.40
wly	12:27	7.30	8.60	7.80	10.00	7.60	11.10	4.30	9.30	7.45	8.35
ylb	16:40	7.80	15.60	14.55	15.30	12.35	12.60	10.55	12.55	12.20	13.10
wwr	14:25	8.50	6.80	9.45	7.30	10.30	8.30	3.80	2.70	6.40	5.00

ow	15:20	9.20	7.50	9.50	7.30	13.20	7.80	13.60	12.50	13.30	8.00
rwb	13:50	8.90	8.10	10.50	8.10	10.10	8.10	10.40	8.50	11.20	8.55
wrl	14:20	8.70	5.10	8.90	8.10	13.00	12.60	16.00	12.80	13.20	8.20
wyb	14:42	6.60	6.80	0.00	8.30	8.20	14.85	14.60	16.60	13.10	14.10
yry	14:45	12.30	11.90	13.70	13.40	16.60	12.90	13.40	12.50	12.90	8.10
wby	14:55	10.00	5.90	9.90	7.90	8.10	7.60	11.60	10.65	11.40	8.30
ybb	15:10	6.50	12.40	12.20	15.30	12.25	12.60	12.10	12.60	12.10	14.50
ywr	13:45	9.20	7.40	9.10	7.40	9.60	8.50	10.30	7.95	9.05	7.90
ywy	14:10	8.90	8.20	9.20	7.30	11.70	9.90	13.85	12.60	13.70	11.70
yww	15:10	15.20	14.80	15.15	14.00	15.30	13.50	14.10	13.80	15.00	14.40
wyr	12:34	7.80	15.00	10.60	15.80	6.80	7.90	7.70	11.10	5.50	6.10
yrl	12:40	8.50	7.60	10.85	8.20	8.65	7.20	7.70	6.40	11.85	11.00
wwl	12:50	9.20	4.50	10.40	8.40	9.25	8.90	12.80	11.25	14.50	13.80
wrb	13:30	8.10	8.50	4.80	7.65	7.10	13.20	12.00	14.80	0.00	1.10

Two-feeder experiment, R-training (left channel end at 0 m; right channel end at 16.6 m)

Ant	Time	1	2	3	4	5	6	7	8	9	10
wrw	11:25	9.30	6.40	11.40	10.65	11.60	11.00	11.50	10.80	11.30	10.80
wyl	12:00	9.50	8.00	11.30	8.90	11.70	6.40	11.55	10.00	13.50	6.50
wlr	12:06	8.85	8.30	9.80	8.40	15.40	14.20	15.30	12.10	15.35	14.30
wrb	13:10	8.00	8.70	8.30	11.70	11.30	11.50	8.60	11.90	5.20	8.50
yby	11:02	9.10	8.60	9.25	8.30	10.00	9.70	10.70	6.70	8.70	6.90
ybr	11:21	8.50	8.10	8.50	7.20	9.90	3.80	8.60	8.00	8.70	8.20
wgg	11:11	7.60	9.70	7.20	9.35	8.55	10.30	8.30	10.00	6.80	12.30
ryr	11:45	9.30	8.20	11.70	6.70	11.30	11.00	15.55	8.80	11.60	9.30
ryy	11:50	9.10	8.90	9.30	7.10	10.30	9.80	11.10	10.40	11.30	7.80
wrb	12:01	8.60	7.90	8.50	7.30	9.20	3.90	4.25	2.50	7.90	4.70
yrw	13:03	11.10	9.45	12.30	9.90	10.40	8.35	12.50	12.00	15.60	13.75
yyy	11:23	8.50	7.90	8.60	7.00	9.10	7.70	9.00	6.80	7.50	7.00
ybw	11:58	10.60	9.50	10.70	7.60	13.20	11.00	14.10	11.60	12.80	10.70
rgy	12:56	8.60	8.00	10.30	7.70	10.80	6.10	9.20	3.70	8.10	2.20

wrl	13:05	9.25	8.00	10.30	7.40	11.60	8.05	8.60	4.65	11.50	4.50
yrb	10:32	8.00	9.30	8.00	8.80	8.20	10.20	8.00	9.80	7.80	9.40
wyb	10:56	7.90	11.50	8.40	16.40	13.20	16.60	13.25	16.60	12.90	13.60
wyr	14:30	8.10	10.00	7.10	12.60	5.10	10.40	4.60	7.10	3.50	4.30
wbr	14:45	9.60	6.80	16.60	12.30	16.55	14.60	15.00	13.80	14.80	6.80
wyw	14:52	8.50	6.30	9.40	6.90	16.00	5.50	9.60	3.90	5.40	4.90
wrr	16:00	7.70	11.40	7.05	15.40	7.20	14.20	13.50	14.30	9.50	11.70
wby	14:19	9.80	7.90	13.70	6.25	14.50	7.30	8.80	8.10	9.70	8.60
wll	15:25	8.10	10.40	7.00	7.40	4.60	11.70	5.30	10.50	9.20	9.50
wrw	14:30	8.10	16.60	16.20	16.60	14.90	16.60	8.00	8.30	8.10	9.30
yyb	15:05	16.50	12.10	16.50	10.25	15.80	9.30	11.75	4.30	7.70	2.80
rrr	15:27	9.10	7.80	10.55	6.50	7.70	5.80	7.80	6.80	8.10	2.20
wbr	10:27	5.90	6.50	4.30	7.50	6.40	8.25	7.80	8.70	7.90	8.30
wrr	10:24	9.00	8.40	9.20	7.20	11.60	10.80	13.40	10.00	10.70	10.10
wrr	10:26	7.90	9.70	7.50	11.40	9.30	13.10	11.80	12.50	9.00	12.95

One-feeder control experiment (right channel end at 0 m; left channel end at 16.6 m)

**Ant**	**Time**	**1**	**2**	**3**	**4**	**5**	**6**	**7**	**8**	**9**	**10**
wbb	10:55	10.60	8.20	8.60	2.20	3.65	0.00	0.20	0.00	0.30	0.00
bww	10:40	7.10	11.00	6.20	8.50	8.30	16.60	16.40	16.60	15.90	16.30
wbl	12:30	7.50	8.70	7.70	10.00	6.00	11.20	5.40	8.30	7.70	9.60
wrb	09:58	8.45	7.80	8.70	5.40	8.30	4.70	9.10	7.00	8.30	8.20
wrw	10:08	7.90	9.10	7.00	9.60	4.10	8.40	8.20	10.40	3.60	5.50
yyy	10:15	7.40	9.10	6.60	9.20	8.20	8.90	8.20	8.40	7.40	8.70
bby	10:49	8.10	12.50	9.60	10.00	8.30	8.60	7.60	9.00	3.00	5.20
yyg	12:23	8.50	7.90	9.50	7.50	8.70	5.90	9.90	7.50	11.25	9.20
bbb	14:18	9.50	6.00	16.60	9.70	11.10	1.00	13.60	10.00	16.60	16.40
www	16:00	8.60	8.20	16.60	15.20	16.60	0.00	15.50	0.00	0.50	0.00
rww	13:54	8.70	6.60	10.30	5.00	9.00	7.60	10.40	8.20	8.60	4.50
www	13:45	10.20	8.30	8.50	7.70	10.50	8.00	9.10	5.60	8.60	8.30
rwg	14:25	9.10	7.60	10.10	7.90	10.10	8.10	8.60	8.10	8.40	8.30

wrr	14:35	8.10	11.00	7.30	11.10	3.60	9.80	7.50	10.90	5.30	11.00
rwb	15:55	7.40	9.70	5.90	10.50	7.50	8.10	5.90	11.90	9.00	10.35
wgg	16:20	7.40	8.40	7.20	9.50	7.60	9.20	5.20	7.50	6.50	10.55
wbw	16:40	7.80	9.30	8.10	8.70	3.30	12.70	9.70	10.40	4.80	8.90
